# Platelet Rich Plasma for Regenerative Medicine Treatment of Bovine Ovarian Hypofunction

**DOI:** 10.3389/fvets.2020.00517

**Published:** 2020-08-13

**Authors:** Fausto Cremonesi, Stefano Bonfanti, Antonella Idda, Anna Lange-Consiglio

**Affiliations:** ^1^Dipartimento di Medicina Veterinaria, Università degli Studi di Milano, Milan, Italy; ^2^Private Practitioner, Milan, Italy

**Keywords:** bovine, ovarian hypofunction, platelet rich plasma, progesterone, growth factors

## Abstract

Recent studies on cull cows have shown that ovarian abnormalities, particularly ovarian insufficiency, are the main cause of reproductive failure. The aim of this study was to treat bovine ovarian failure with intraovarian administration of autologous platelet rich plasma (PRP), which is rich in growth factors, chemokines, and cytokines that could stimulate follicular growth and steroidogenesis. Twelve cows with ovarian hypofunction were enrolled in the study and they were randomly allocated in control group (CTR) and treated group (six animal for group). In the treated group, only five animals received the PRP treatment because intraovarian administration was hindered in one by a rectovaginal fistula. Animals of control group were treated by intraovarian administration of physiological solution. In the 4 weeks after PRP injection, a mild to strong increase in progesterone (PRG) concentrations was detected in four of the five cows treated. Artificial insemination (AI) resulted in four pregnancies that are still ongoing (7th month). Intraovarian administration of PRP improved ovarian function after 2 months of treatment. This effect may be due to reduction of follicular atresia or to revitalization of dormant oocytes allowing restoration of fertility.

## Introduction

In recent years, the decline in fertility is negatively related to the increase in milk production in dairy cattle and it is common worldwide. Indeed, to date there is a large scientific literature on the sub-fertility of dairy cows (1). Recent studies showed that the main causes for low fertility in dairy cows are reproductive disorders (2), mainly due to ovarian abnormalities. There are different kind of ovarian disorders such as ovarian hypofunction, cystic ovarian disease, sub-oestrus, or silent ovulation, and sub-luteal function. Although all ovarian disorders have an impact on profits, and are a challenge for the dairy manager, this paper focuses on ovarian hypofunction and ovarian failure. Within the different ovarian disorders, ovarian failure is the most common ovarian disorder (11.45%) compared to cysts and adhesions (5.22 and 6.38%, respectively) (3).

Ovarian hypofunction is determined by the presence of follicles with a diameter of at least 8–15 mm visible in two consecutive examinations, by the absence of a corpus luteum or cysts without signs of oestrus in the 7-day period between the examinations (4).

During the bovine oestrous cycle, the development of antral follicle occurs through two or three waves of follicular growth (5, 6). Follicles of 3–4 mm diameter grow to about 6–8 mm in diameter. When the largest follicle reaches a diameter of 8.5 mm (7), it begins its follicular dominance that determines its size increment and the arrest of the smaller subordinate follicles. The dominant follicle will become the ovulatory follicle (8) and the luteinizing hormone (LH) appears to regulate its function (9, 10).

Rapid LH pulse frequency (1 pulse/15–20 min) is correlated with the maturation and ovulation of the dominant follicle, on the contrary, slow LH pulse frequency (1 pulse/3–5 h) results in follicular atresia (11).

Ovarian hypofunction can be related to intermediate LH frequencies (1 pulse every 1–2 h) due to different factors such as low progesterone concentrations or heat stress (1). Usually, these cows have a follicle of at least 8–15 mm and absence of cysts or luteal structures on two consecutive examinations during the postpartum period, so it can be assumed that most cows affected by ovarian hypofunction have ovarian anovulation.

Currently, these cows would be treated with gonadotropin releasing hormone (GnRH)-based treatments such as the Ovsynch protocol (4, 12, 13) or a progesterone releasing intravaginal device (PRID) (14), but oestrus response in affected cows is very low (usually <30%). There is clearly a need for better reproductive strategies to reduce the economic impact of decreased fertility. These strategies should increase the amount of cyclic cows and the effectiveness of oestrus detection but most importantly, improve ovarian function and completion of follicle growth. Since the blocking of follicular growth results in a low positive feedback to the hypothalamus, an alternative strategy for treatment of ovarian failure could be an administration of regenerative elements directly into the ovary, with the aim of stimulating follicular growth and, consequently, steroidogenesis in turn stimulating gonadotropin (FSH and LH) secretion.

This study proposes a novel therapeutic approach for treatment of ovarian failure based on the regenerative properties of PRP already well-documented in human (15, 16) and veterinary (17, 18) medicine.

## Materials and Methods

The *in vivo* animal study was approved by the Milan University Bioethics Committee n.118_2017, in accordance with 2010/63 EU directive on animal protection and Italian Law (D.L. No. 116/1992) and following standard veterinary practice. An authorization number 658/2020-PR of the Italian ministry of health has also been obtained. Written informed consent from the owners was also obtained to allow evaluation of the *in vivo* effects of PRP in cows with ovarian failure.

### Study Design

To investigate the effect of PRP in restoring ovarian function in cows with ovarian failure, the study was divided into two parts: (1) *in vitro* production of PRP; (2) *in vivo* intraovarian PRP administration.

### *In vitro* Production of PRP

Homologous platelet rich plasma was prepared from blood collected from animals enrolled in the study. To produce PRP, the blood was processed as described by Lange-Consiglio et al. (17). Briefly: the site of blood collection was the subcutaneous mammary vein. Blood (450 ml) was collected in *ad hoc* Terumo blood bags (Terumo Srl, Rome, Italy), containing CPDA-1 and transported at +4°C to the laboratory to be immediately processed.

In aseptic conditions, the blood was drawn into sterile 50 ml Falcon tubes (Euroclone, Milan, Italy) and centrifuged at 100 × g for 30 min. The result of centrifugation was the separation of the blood into three components: red blood cells at the lowest level, “buffy coat” in the middle layer, and platelet-rich plasma (PRP) in the upper layer. The PRP was aspirated and centrifuged again at 1,500 × g for 10 min to obtain a platelet pellet and the platelet poor plasma (PPP). The pellet was mixed in a PPP volume to obtain a PRP at a standard concentration of 1 × 10^9^ platelet/ml. All platelet counts on peripheral blood and PRP were performed by an automatic hematology analyzer HeCo Vet SEAC (Florence, Italy).

The total volume of PRP obtained from each animal was stored in syringes ready-to-use. The syringes were frozen at −80°C and thawed three times at 37°C (19) to allow the release of platelet derived factors. Syringes containing the dose of 5 ml of PRP were kept frozen at −80°C until use. The PRP was subjected to bacteriological examination to verify its sterility.

### *In vivo* Intraovarian PRP Administration

#### Animals

Animals enrolled in this study belonged to the same livestock and their welfare conditions (related to housing, feeding, and watering) were identical to those of the remaining animals of the same farm.

This farm had an endowment of about 1,500 animals, with a conception rate of 32% and pregnant rate of 19%. The ovarian hypofunction affected about 10% of adult cows and, usually, it was treated with the Ovsynch protocol with oestrus response about of 25%. The average milk production of farm was of 34 l/die while the average of the animals affected by ovarian hypofunction was of 38 l/die. The animal affected by this pathology were more productive of the other animals.

Twelve Holstein–Friesian cows, 2–3 years old, with no oestrus signs after the voluntary waiting period for breeding (around 60 days), were monitored via cow podometers for two oestrus cycles before being diagnosed as anoestrus. Ultrasound examination (SonoSite Portable ultrasound with a 5.0 MHz linear-array transducer, Inc, USA) confirmed bilateral ovarian failure for all the subjects enrolled in the study. The animals were randomly allocated in treated group with intraovarian administration of PRP and in control group (CTR) with intra-ovarian administration of placebo to confirm that the needle does not cause trauma to the ovary. Each group was composed of six animals.

#### Setting

In this farm, the lactating cows were housed in bunks with a concrete floor but with the availability of clean straw. The barn was clean, airy, well lit, with shaded areas, fans, and watering facilities. An automatic control system was used to remove dung four times a day (at 6.00, 11.00, 18.00, and 23.00 h). Lactating cows were fed twice a day during the experiment period (at 5.00 and 16.00 h) and received total mixed ration composed of medical bandaged, medical dry, soybean flour, barley, cotton, and cornmeal. This mixed ration was supplemented with milk and silage. Mineral salt and water were provided *ad libitum*.

#### Intraovarian Injection of PRP or Placebo

In the treated group animal, the intraovarian PRP injection was performed under ultrasound guidance using a Madison SA 600v instrument equipped with an ovum pick up probe (PB-06VE65/20BD) as described by Cremonesi et al. (20). Each animal was restrained in a cattle cage and clipped over the S5-C1 vertebrae (5 × 5 cm). The area was cleaned three times with alcohol and povidone-iodine. Then, sacrococcygeal (SC) epidural anesthesia was induced with 4 ml of Procaine hydrochloride 2% (Procamidor, Richter Pharma Ag) and confirmed by loss of tail tone. Before introduction of the ultrasound probe into the vaginal fornix, the vulva was cleaned and the vagina lubricated. The ovary was manually directed to the probe by rectal palpation. A spinal 18G needle connected to a steel tube was inserted into the probe needle guide and passed through the fornix to enter the ovarian stroma to administer the PRP. Five cows received 5 ml of PRP with a concentration of 1 × 10^9^ platelet/ml in each ovary. A rectovaginal fistula in the sixth cow prevented access to the ovaries and no PRP was administered in this cow.

The dose of PRP administered was calculated following the procedures of Pantos et al. (21) and by tests carried out in our laboratory with bovine ovaries recovered at the slaughterhouse.

In the control animal, the ovaries were injected with 5 ml of placebo (physiological solution: 0.9% NaCl) with the same protocol of treated animals.

#### Data Measurement

A blood sample (10 ml) was collected from the tail vein of each cow before PRP or placebo treatment (week 0) to measure progesterone (PRG) concentrations. Blood analysis was repeated after 2 and 4 weeks of treatment. The blood was refrigerated at 4°C for 30 min. The upper serum was then collected by centrifugation for 10 min (3,000 × g) and placed in a −80°C freezer. The plasma concentration of progesterone was assessed using a quantitative automated method based on the enzyme-linked fluorescent assay (ELFA) technique (Mini-Vidas; bioMérieux Italia S.p.A., Florence, Italy).

**Figure 1:**
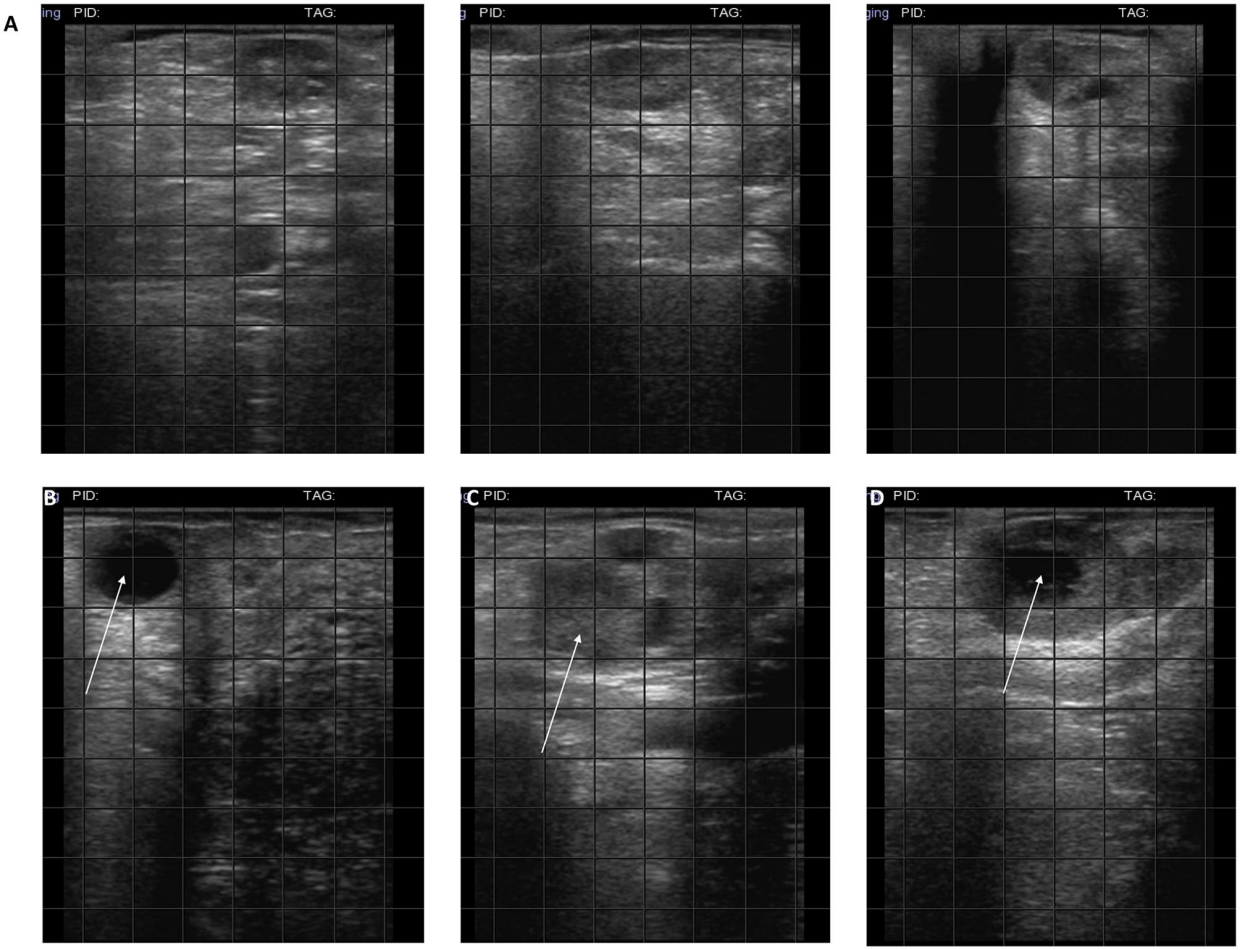
Ultrasound of ovaries. **(A)** Hypogonadal ovaries; **(B)** the arrow indicates 2 cm follicles; **(C)** the arrow indicates a corpus hemorrhagicum; **(D)** the arrow indicates a corpus luteum.

In the weeks following treatment, ovaries were monitored by ultrasound and follicles or corpus luteum were detected in all treated cows (Figure 1) but not in control animals.

Breeding experiments were performed when animals were detected in oestrus. Artificial insemination, with cryopreserved semen thawed for 30 s at 36–37°C and evaluated for quality was performed at 8–12 h after signs of oestrus. Frozen semen from the same bull of proven fertility was used for artificial insemination (AI) in all cows.

Pregnancy diagnosis was performed by ultrasound at 38, 56, and 120 days.

**Figure 2:**
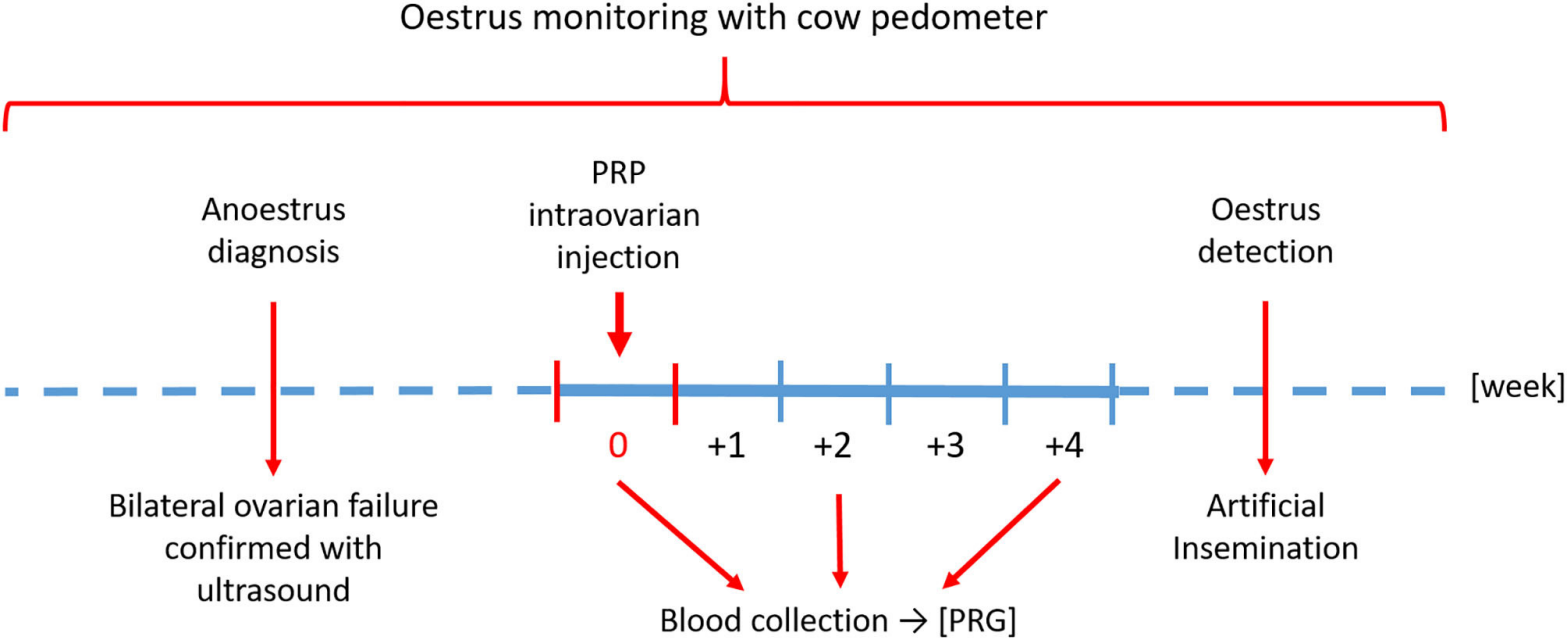
Timeline of the study.

The timeline of the study is shown in Figure 2.

### Statistical Methods

Progesterone concentration data were analyzed with RStudio Version 1.2.1335. Data normality was evaluated with Shapiro–Wilk Normality Test. For non-normally distributed data, Kruskal–Wallis non-parametric test was used. Differences were considered statistically significant when *p* ≤ 0.05.

## Results

Only five animals of treated group were treated with intraovarian administration of PRP because a rectovaginal fistula in one cow prevented access to the ovaries.

In the 4 weeks after PRP injection, a mild to strong increase in progesterone concentration was detected in four of the five cows treated (Table 1). No variation in progesterone concentration was detected in CTR animals (Table 2).

**Table 1:** Dataset showing identification information for each treated cow enrolled in the study (ID, age, lactation) and progesterone concentration and pregnancy detection after intraovarian PRP injection.

**Cow ID**	**Age (years)**	**N^**°**^ lactation**	**Progesterone concentration (ng/ml)**			**Pregnancy after PRP treatment**
			**week0**	**week+2**	**week+4**	
70	3	2	0.89	1.57	4.27	Yes
175	2	1	0.32	0.31	3.54	Yes
424	4	3	0.45	2.63	0.58	No
438	3	2	1.87	1.14	6.23	Yes
779	5	4	Not treated because of rectovaginal fistula			
1,078	2	1	1.2	5.67	4.76	Yes

**Table 2:** Dataset showing identification information for each control cow enrolled in the study (ID, age, lactation) and progesterone concentration and pregnancy detection.

**Cow ID**	**Age (years)**	**N^**°**^ lactation**	**Progesterone concentration (ng/ml)**			**Pregnancy**
			**week0**	**week+2**	**week+4**	
321	2	1	0.47	0.65	0.82	No
267	2	1	0.45	0.75	0.69	No
643	3	2	0.37	0.66	0.84	No
185	4	3	0.77	1.03	0.82	No
482	3	2	0.86	0.66	0.49	No
947	3	2	0.49	0.79	0.85	No

After 4 weeks, in cow number 70 the progesterone increased 4.8 fold (from 0.89 to 4.27 ng/ml). In cow 175, the increase was 11 fold (from 0.32 to 3.54 ng/ml). In cow 424, the progesterone concentration remained unchanged (0.45–0.58 ng/ml). In cow 438, the progesterone increased 3.33 fold (1.87–6.23 ng/ml) and in cow 1,078, there was a 3.97 fold increase (1.2–4.76 ng/ml).

Despite the numerical increases, there were no statistically significant differences (*p* > 0.05) in progesterone concentrations between week 0 and 4.

**Figure 3:**
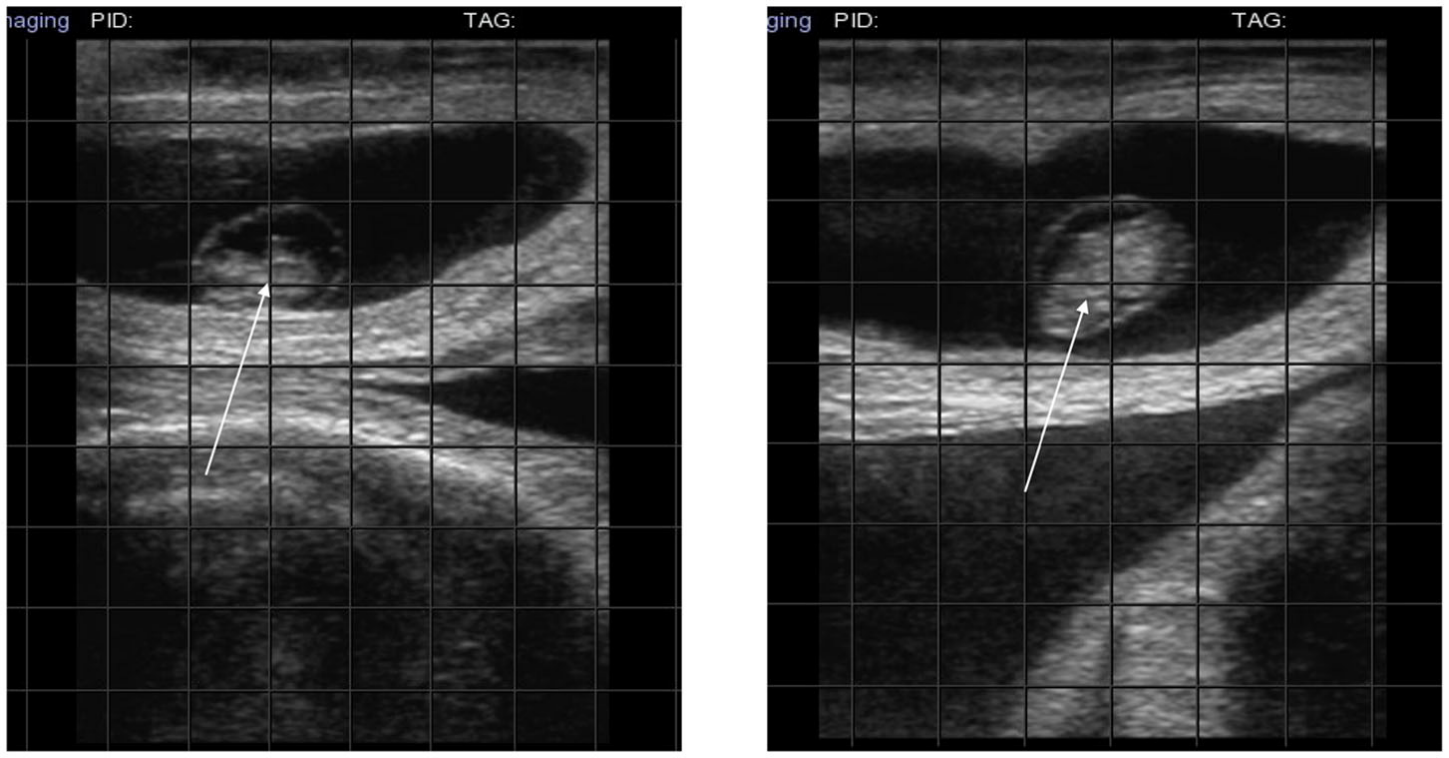
Ultrasound at 38 day. The arrows indicate the embryo confirming pregnancy.

In four cows in which PRP administration was followed by an increase in progesterone concentration, AI resulted in a pregnancy that is still going (7th month) (Figure 3, diagnosis at 38 days).

## Discussion

This study describes the administration of intraovarian PRP to treat cows diagnosed with ovarian failure at the end of the voluntary waiting period after their last parturition. One cow was excluded from the study due to a rectovaginal fistula that hindered the PRP injection.

The rational for application of PRP to hypo-functional ovaries comes from its known properties in regenerative medicine. Indeed, PRP is mainly used in human medicine to accelerate the healing process following surgery (for example maxillofacial surgery), in the repair of muscle and/or tendon, and in the reversal of skin ulcers (16). In veterinary medicine, the therapeutic use of PRP is still very limited. It has been mainly used to promote equine tendon repair (22), to treat intestinal wound healing in pigs (23), to cure a large cutaneous lesion in a dog (24), in bovine mastitis (17) and in repeat breeder cows (18). In all cases, PRP showed a clear regenerative potential due to its amount of growth factors with mitogenic and anti-inflammatory potential (16, 25). In particular, this hematic product can be defined PRP only if it has a platelet concentration 3–5 fold higher than the physiological level. The PRP contains several growth factors including the transforming growth factor β1 (TGF-β1) and TGF-β2, platelet derived growth factors (PDGFAA, PDGF-BB, and PDGF-AB), insulin-like growth factor 1 (IGF-I), epidermal growth factor (EGF), vascular endothelial growth factor (VEGF), fibroblast growth factor (FGF), and hepatocyte growth factor (HGF). All these factors, which are stored in the α-granules, are very important in the regeneration process. The α-granules release their content following their breakdown which can occur physically by temperature excursion (19) or by induced platelet activation (26).

The regenerative effect of PRP in the reproductive field was first muted by the Pantos group, which was able to stimulate ovarian rejuvenation and obtain livebirths in peri-menopausal women with infertility (27). These promising results were expanded by Sills et al. (28), who performed intraovarian administration of PRP in a clinic setting, without anesthesia, using transvaginal ultrasound guidance, and confirmed by Pantos et al. (21). These studies reported an increase in serum AMH, decrease in FSH, fertilization via intracytoplasmic sperm injection of collected oocytes, and generated blastocysts suitable for banking or immediate embryo transfer. Pregnancies were also reported (21, 28). The impact of PRP in treating human reproductive disorders is also reported in some papers describing its positive effect after intrauterine administration in patients with repeated implantation failure (28) and chronic endometritis (29).

In the veterinary field, a study performed by our team showed that PRP induced an increase in progesterone receptors after *in vivo* uterine administration in healthy cows and exerted a modulatory effect on a range of molecules involved in inflammation, which resulted in an anti-inflammatory effect in an *in vitro* endometritis model (30).

During development, the ovary faces a variety of changes and remodeling. The process of follicular growth begins from the original oocyte that derived from primordial germ cells (31). During embryogenesis, the ovarian cells of the female fetus are delimited by granulosa cells to form primordial follicles (32). Granulosa cells are necessary during follicular development (33, 34) and follicular atresia is probably mainly caused by granulosa cell apoptosis.

Presumably, the PRP effect on ovarian dysfunction is due to secretion of nutritional factors that produce beneficial effects on follicles. To date, the mechanism of action on the ovary is not entirely clear, but there are two main hypothesis that refer to ovarian reserve. It is well-known that, at birth, the mammalian female has a fixed ovarian that is dormant and closed in primary follicles until the moment of activation, when growth and meiotic occur. The first hypothesis suggests that growth factors and cytokines in PRP act on dormant oocytes exerting a revitalizing effect and restoring fertility (28).

The other hypothesis supports the existence of ovarian germ cells from which would be generated new oocytes (28). The existence of ovarian stem cell and the detailed study of the mechanisms underlying the PRP action is worthy of further study, but goes beyond the scope of our preliminary work in this animal model.

This study focused on empirical assessment of the efficacy of PRP through measurement of progesterone (PRG) levels as a marker for restored ovarian cycling and the monitoring of oestrus to perform AI. Data reported in this study are certainly limited but do support recent reports on the capacity to restore physiological function of a malfunctioning ovary using PRP.

In particular, our data show that after intraovarian PRP injection (week 0), the level of PRG increased between 3 and 11 fold during the subsequent 4 weeks (Table 1). This increase in PRG concentration indicates recovery of ovarian cycling. Presumably, in light of the large changes in PRG measured, each cow had more than one ovulation during those 4 weeks. Artificial insemination was performed when oestrus monitoring signaled the heat. Artificial insemination was needed on more than one occasion to obtain a pregnancy. As indicated in the right column of Table 1, the time between PRP injection and the detection of pregnancy varied for each cow and depended on the success of AI. Pregnancy occurred only in the four cows that experienced increased PRG levels. Only one animal failed to respond to PRP treatment. Further studies including larger numbers of cows are required in future studies.

No analysis of serum AMH or FSH levels was performed. This could have added more accurate information on the temporal changes in the cows' hormonal status but it is likely that a rise in PRG corresponds to a decrease in FSH whilst, to date, there are conflicting reports on the variable levels of AMH in literature (35).

Usually, in this farm, the hypogonadism affects the 10% of animals that are treated with the Ovsynch protocol, with oestrus response about of 25%. In our study, we had performed a different control group with intra-ovarian administration of placebo to confirm that it was not the trauma caused by the needle that awakened the ovarian activity but really the PRP. In our opinion, the main platelet-derived mediators in improving ovarian could be the PDGF, the TGF-β, and the HGF because all these growth factors have mitogenic or trophic effects and, specifically, the TGF-β that can stimulate cell proliferation and differentiation. In this moment, it is no possible to affirm that the ovarian activity is restored in the long time by PRP because the four treated cows are pregnant and only after the delivery and the voluntary waiting period for breeding, the oestrus signs will be evaluated to confirm the efficacy of PRP in this pathology.

## Conclusions

This is the first report of successful pregnancies after PRP intraovarian injection in cows with ovarian failure. These findings support the use of PRP in clinical practice, supplement the literature regarding the use of PRP in a reproductive context, and motivate the study of the fine mechanisms underlying PRP action.

## Data Availability Statement

The raw data supporting the conclusions of this article will be made available by the authors, without undue reservation.

## Ethics Statement

The animal study was reviewed and approved by Milan University Bioethics Committee no. 118_2017 and by authorization number 658/2020-PR of the Italian ministry of health. Written informed consent was obtained from the owners for the participation of their animals in this study.

## Author Contributions

FC: conceptions and design, *in vivo* study with administration of PRP, evaluation of outcomes, interpretation of data, and final approval of manuscript. SB: *in vivo* study with administration of PRP, evaluation of outcomes, interpretation of data, and final approval of manuscript. AI: preparation of PRP, analysis and interpretation of data, and final approval of manuscript. AL-C: conceptions and design, preparation of PRP, coordination of all experiments, collection and assembly of all data, analysis and interpretation of data, manuscript writing, and final approval of manuscript. All authors contributed to the article and approved the submitted version.

## Conflict of Interest

The authors declare that the research was conducted in the absence of any commercial or financial relationships that could be construed as a potential conflict of interest.

## Abbreviations

AI: artificial insemination
EGF: epidermal growth factor
FGF: fibroblast growth factor
GnRH: gonadotropin realizing hormone
HGF: hepatocyte growth factor
IGF-I: insulin-like growth factor 1
LH: luteinizing hormone
PDGFAA, PDGF-BB, PDGF-AB: platelet derived growth factors
PRG: progesterone
PRID: progesterone releasing intravaginal device
PRP: platelet rich plasma
TGF-β1: transforming growth factor β1
TGF-β2: transforming growth factor β2
VEGF: vascular endothelial growth factor.
